# Analysis of Caregiver Burden Expressed in Social Media Discussions

**DOI:** 10.3390/ijerph20031933

**Published:** 2023-01-20

**Authors:** Catherine C. Shoults, Michael W. Rutherford, Aaron S. Kemp, Merideth A. Addicott, Aliza Brown, Carolyn J. Greene, Corey J. Hayes, Jennifer M. Gan, Linda J. Larson-Prior, Jonathan P. Bona

**Affiliations:** 1 Department of Biomedical Informatics, University of Arkansas for Medical Sciences, Little Rock, AR 72205, USA; 2 Department of Psychiatry, University of Arkansas for Medical Sciences, Little Rock, AR 72205, USA; 3 Department of Physiology and Pharmacology, Wake Forest University School of Medicine, Winston-Salem, NC 27157, USA; 4 Department of Neurology, University of Arkansas for Medical Sciences, Little Rock, AR 72205, USA; 5 VA HSR&D COIN Center for Mental Healthcare and Outcomes Research, Central Arkansas Veterans Healthcare System, Little Rock, AR 72205, USA; 6 Department of Medical Humanities and Bioethics, University of Arkansas for Medical Sciences, Little Rock, AR 72205, USA; 7 Department of Neurobiology and Developmental Sciences, University of Arkansas for Medical Sciences, Little Rock, AR 72205, USA

**Keywords:** caregiver burden, social media data mining, spillover effects

## Abstract

Almost 40% of US adults provide informal caregiving, yet research gaps remain around what burdens affect informal caregivers. This study uses a novel social media site, Reddit, to mine and better understand what online communities focus on as their caregiving burdens. These forums were accessed using an application programming interface, a machine learning classifier was developed to remove low information posts, and topic modeling was applied to the corpus. An expert panel summarized the forums’ themes into ten categories. The largest theme extracted from Reddit’s forums discussed the personal emotional toll of being a caregiver. This was followed by logistic issues while caregiving and caring for parents who have cancer. Smaller themes included approaches to end-of-life care, physical equipment needs when caregiving, and the use of wearables or technology to help monitor care recipients. The platform often discusses caregiving for parents which may reflect the age of Reddit’s users. This study confirms that Reddit forums are used for caregivers to discuss the burdens associated with their role and the types of stress that can result from informal caregiving.

## 1. Introduction

The role of caring for individuals suffering from chronic disabling conditions often falls to a family member. In 2013, an estimated 39% of US adults provided unpaid, non-professional care to a loved one, up from the 27% estimated in 2010, and this percentage is expected to rise further as the population ages [1,2,3]. As the provision of long-term services and support has shifted from formal healthcare providers to family members and the community, the importance of family caregivers has been increasingly recognized, with a national profile of family caregivers first emerging in the 1997 *Caregiving in the U.S.* study, most recently updated in 2020 [4]. Caregiving is an activity that occurs in all generations, ethnic identities, gender identities, and socioeconomic levels, with data indicating that many caregivers are taking on their role with inadequate support [4].

The concept of caregiver burden was first proposed in 1966 and has since been recognized as a complex multidimensional concept that includes both the positive and negative components of providing care [5,6], encompassing the uniquely emotional, physical, social, and economic aspects for individual caregivers [3,7,8]. The recognition of the need to understand the impact of caregiving on unpaid family providers has engendered an exponential growth in the literature on caregiving since about 2000 [9]. In this literature, the majority of studies rely on cross-sectional survey data [3,7,10], focusing largely on a single care recipient condition such as neurological [11,12,13,14], cancer [15,16,17], or neuromuscular [18,19,20] disorders. However, an increasingly large amount of the literature is devoted to meta-analytic or systematic reviews [21,22,23,24] that generally assess the burden across multiple types of caregivers and care recipient conditions. While the extent of the burden and the specific emotional, physical, and socioeconomic aspects considered to be most burdensome differed between studies, overall, the literature supports the current view of caregiving as a complex, multidimensional, and individually unique issue.

While there are studies that have relied on structured interviews with familial caregivers [25,26], we are aware of only one that has utilized social media to gain insight into caregiving concerns: Cooper (2021) [27] utilized caregiver narratives derived from three online platforms that invited users to share their experiences in a free-form narrative, with a focus on examining the online identities family caregivers build in their narratives. In this study, we leveraged public content posted pseudonymously by users of the social media platform Reddit (Reddit.com), which can provide unique insights into user’s lives and opportunities to understand the views and concerns of individual caregivers through support forums focused on caregiving. Pseudonymous discussions on Reddit forums are publicly accessible and can be readily collected and analyzed in aggregate. Because Reddit uses topic-labeled communities/forums that allow users to feely post textual content of any length that community members in the forum can further discuss, and because it includes several forums specifically focused on caregiving, it is an ideal social media platform from which to gather information on the perceived burdens and concerns of familial caregivers. The use of support groups has previously been suggested to operate as a psychosocial intervention for caregivers [28], making online support forums a potential alternative to in-person support groups.

This study examines public social media discussions on Reddit by individuals engaged in informal caregiving. Specifically, our aims were to (1) extract the themes of the caregiver burden from informal narratives found on Reddit caregiver communities using Natural Language Processing (NLP) and (2) interpret those caregiver burden themes using an expert insight to the NLP results and interactive visualizations. The overall goal of this line of research was to identify the specific unmet needs of caregivers as a first step towards providing targeted caregiver support.

## 2. Materials and Methods

**Figure 1:**
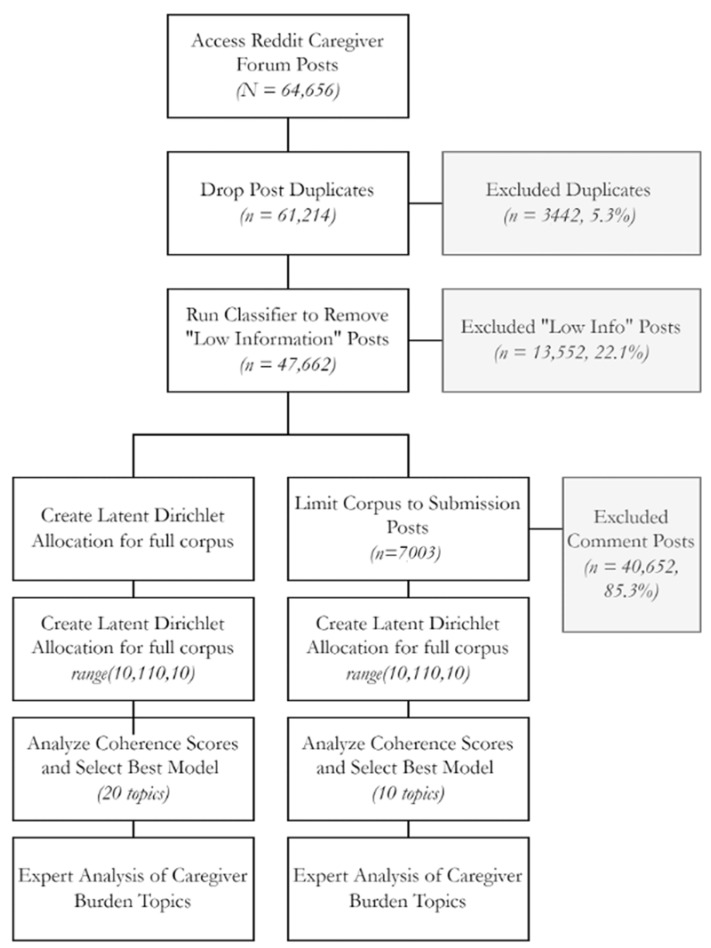
Methodology flowchart from accessing Reddit posts through creation of Latent Dirichlet Allocation for two corpuses (full Reddit dataset and Submissions-only dataset).

Publicly accessible posts (2008–2022) from Reddit.com caregiver discussion forums were programmatically collected using the Pushshift.io application programming interface (API) which was accessed on 21 Jun 2022 [29] and custom scripts were implemented in Python version 3.8.5. Posts were collected from five Reddit discussion forums: r/cancercaregivers, r/caregivers, r/caregiversofreddit, r/caregiving, and r/caregiversupport. The API was queried from both submissions and comments (collectively called “posts”). Using the Python Natural Language Toolkit (NLTK) [30], text was preprocessed through tokenization, stopword removal, and lemmatization. Figure 1 provides the flowchart and corresponding number of post counts for each step in the process.

A machine learning classifier was created to remove “Low Information” posts or posts that did not contain insights into the caregiver experience. Examples include spam posts advertising for a medication or other product, posts containing short replies with no real content (e.g., “Ok, thanks!”), and similar. These were removed because they clearly provide no insight into the caregiver experience. Two annotators independently reviewed a random sample of 2000 posts (Cohen’s Kappa = 76.5%). In total, 1600 posts were used to create a classifier using Gradient Boosted Classification. Two classifiers were created: one using the cleaning methods used above (tokenization, stopword removal, and lemmatization) and one without text cleaning (raw text). Raw text achieved a better performance (Recall: 0.80, Precision: 0.82, F1: 0.81) and was used to remove low information posts. 

**Figure 2:**
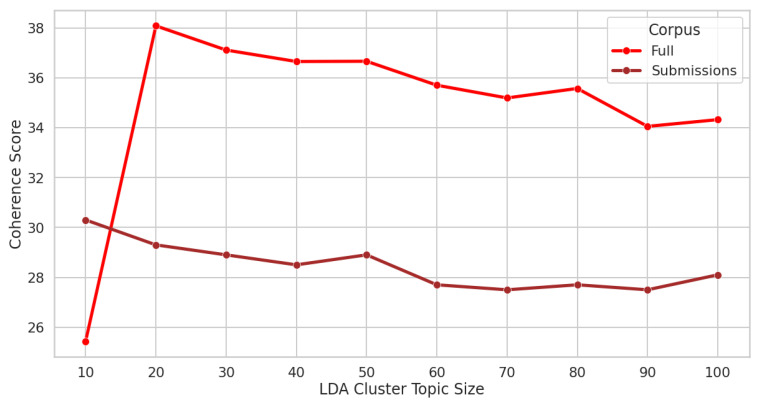
Coherence plots for determining LDA topic size hyperparameter in the Full Reddit dataset (Full) and the Submissions-only dataset (Submissions).

Themes from the text were extracted using the unsupervised machine learning model Latent Dirichlet Allocation (LDA) [31], which automatically extracts “topics”, conceptualized as distributions over all the words contained in a collection of documents. Each document then can be viewed as a distribution over these topics. LDA topic modeling was performed on two corpuses: our entire corpus of Reddit, both submissions and comments in caregiver forums (*n* = 47,662), and a corpus of just Reddit submissions in those forums (*n* = 7003). The hyperparameter tuning of the corpuses was achieved using coherence scores. To find the optimal number of topics, ten different models were built and evaluated using different numbers of topics in 10-topic increments from 10 to 100. The topic cluster with the highest coherence score showed the most similarity between the theme’s words and the topic cluster’s corpus (see Figure 2). 

Interactive visualizations were generated using both pyLDAvis, a Python package for visualizing LDA topics, t-distributed Stochastic Neighbor Embedding (t-SNE) dimensionality reduction, and the interactive visualization library, Bokeh. Themes were created by expert, independent reviewers (*n* = 6) who were provided with the LDA results and two visualizations. The expert reviewers independently assigned labels to the clusters and a final adjudicator looked for similarity in their themes. 

This research was determined as not human subjects research by the University of Arkansas for Medical Sciences (UAMS) Institutional Review Board (IRB 261287).

## 3. Results

**Figure 3:**
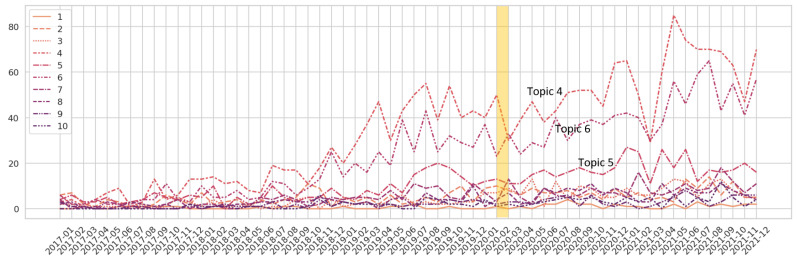
Topics over time showing only 2017 through 2021, although the corpus included low-level response in years prior to 2017. The yellow bar shows when COVID-19 was named by the World Health Organization.

In total, 64,656 posts were downloaded from the API pushshift.io. After removing posts that were classified as “low information”, 47,662 posts were used in the analysis. Analyzing the full corpus, ten LDA machines were created with the hyperparameter cluster size varying from 10 to 100 clusters at intervals of 10. Analyzing the themes from the full corpus, 20 topic clusters had the highest coherence score. When analyzing only the submissions (7003 posts), 10 topic clusters had the highest coherence score. Comparing the full corpus to the submissions-only corpus showed that it was easier to extract the theme from LDA from the submissions-only corpus. In addition, the t-SNE clusters were better differentiated on the submissions-only corpus (see Appendix A for an example t-SNE plot and pyLDAviz plot). The largest topic in this corpus (topic 4 in Table 1 below) discussed the emotional toll of being a caregiver with special emphasis placed on the time burden. This topic showed increased growth starting around the end of 2018 (see Figure 3). 

The second largest topic cluster (topic 6) discussed the logistics of caregiving for a parent, including the power dynamics of the situation. A separate cluster discussed the care of parents who are undergoing cancer treatment (topic 5). Topic 9 focused on the physical equipment needs of caregiving at home, including requests for how to deal with patients who could no longer assist themselves. Spousal care was discussed in topic 8, with mentions of issues such as being employed and having to take time off work to care for their spouse. End-of-life care constituted its own cluster (topic 7). A distinct theme was thankfulness for having the Reddit forum to use as support, although comments of this nature this were found throughout the corpus. Finally, there was a small but distinct topic discussing what technology or wearable devices would help caregivers with their wards (topic 1). 

Figure 3 shows the growth of caregiver-related posts over time, with each line representing a different theme. Caregiver-related posts showed the greatest increase, starting in 2018. The themes held steady over time, with some growth apparent in 2021. Notably, the COVID-19 pandemic did not appear to affect the number of posts about caregiving, though the emerging literature has observed that caregivers were placed under an increased burden during the pandemic [32,33,34].

## 4. Discussion

In this study, we identified ten themes related to caregiver experiences discussed in open access posts on the social medial platform Reddit. These themes broadly cover the perceived burdens related to time management (topics 4, 5, 6, 8), resources and support services (topics 1, 2, 9, 10), and cross-generational caregiving (topics 5, 6). While only topic 4 specifically focused on the emotional burdens related to caregiving, individual posts often referred to feelings of emotional distress, including fatigue, depression, stress, and an inability to cope. Interestingly, in this study, financial burdens were not specifically noted and did not comprise any of our identified topic areas of concern to these caregivers.

In a study focused on the analysis of free-form narratives of personal caregiving experiences using thematic narrative analysis [11,12], the authors identified four unique caregiver identities that provided insight on the negative and positive aspects of caregiving [27]. These included two groups who perceived caregiving as highly burdensome and two other groups who perceived their role in more positive terms. As previously noted, the dichotomy between the negative and positive impacts of caregiving are the frequently reported aspects of the multidimensional concept [3,7,8]. In our study population, posts were overwhelmingly focused on the negative aspects of caregiving and frequently asked others to assure them that the feelings they expressed were not unique but shared by others. 

Family caregivers perform a wide range of tasks with differing levels of effort, from providing companionship, to assistance with activities of daily living, at-home nursing care, and the coordination of treatment and emotional and psychological support [7,35]. Many studies have noted the adverse impact of caregiving on mental and physical health [3,8,36], particularly in cases where the number of caregiving tasks, and the time necessary to accomplish them, increases [35,36,37,38]. The literature indicates that the perceived burdens are higher in those engaged in caring for people with multimorbidity and neurodegenerative disorders [38,39]. In many instances, the time spent in caregiving reduces the time available to the caregiver for social participation and self-care [40,41,42], further adding to the emotional burdens identified in this topic. In addition, studies examining the impact of the COVID-19 pandemic report increases in the caregiver burden and social isolation [39,40].

Time management takes many forms that may include balancing work, family, and caregiving responsibilities, the management of transport to and from medical appointments, and assisting in medication or therapeutic regimes [43]. In this study, the topic of time burden crosses several topic areas and is specifically noted in topic 4, where the emotional toll of time commitments in caring for a family member is explicitly discussed. Care recipients who are less able to perform activities of daily living, who require higher levels of nursing care, or who are at the end of their lives have been previously reported to increase the emotional and physical burdens felt by their caregivers [25,38,44,45], with many caregivers reporting increased stress related to the number of emergency department visits and hospitalizations. Thus, the amount of time spent in caregiving is also related to the increased emotional burdens reported by caregivers.

Increased life expectancy and the increasing number of older adults with multiple chronic diseases is anticipated to result in an increase in family caregivers, with most caregivers caring for a parent or parent-in-law, spouse or partner, grandparent or child. While the majority of caregivers are between the ages of 50 and 64, one report notes that 47% of caregivers are between the ages of 18 and 49. Parental caregivers are often either currently working, caring for children in addition to the parent, or less commonly completing their education [4]. A recent review looked specifically at the impact of caregiving on young, including subadult, caregivers [46] identifying feelings of anxiety, depression, and resentment as negative impacts of caregiving in this population as well as frustration with their lack of knowledge about their loved-one’s condition, decreased time to dedicate to themselves and their personal and professional well-being, and a perception of ‘other’, meaning being different from their peers. Specific to caregiving for a parent with cancer, a recent study compared the burdens reported by adult-children caring for parents with cancer, finding that those caring for a parent experienced greater social, emotional, and financial burdens than did spousal caregivers despite spending less time with their care recipient than did spousal caregivers [47]. That this topic was specifically seen in this study may reflect the demographics of Reddit. While Reddit does not collect information of user demographics, a 2021 Pew survey found that almost 40% of Reddit users are below the age of 30 [48]. While the overall age of Reddit users is young, the users of Reddit caregiver forums may skew older, in which case the discussion of parents may reflect the aging of the baby boom generation (1946–1964) [49].

Finally, we note the broad topic of the need for information, resources, and support services in the caregivers sampled in this study. Posts relating to this topic area in our study indicate the need to better understand the resources that are available and how to apply for assistance, and the need for professional assistance with caregiving chores that the family caregiver feels ill-prepared to provide. The need to provide informational assistance to caregivers has been recognized for over a decade [50] and can include skills training, counseling, social support, and information on local resources that may include respite services or professional support services. Such efforts have included national programs such as the AARP initiated Caregiver Advise, Record, Enable (CARE) Act, which was intended to integrate caregivers into the healthcare team, as those requiring post-hospitalization care were discharged from the formal healthcare system. The success of this program was evaluated in a recent study of its implementation in Pennsylvania [51], finding that the program had been generally successful, although procedural barriers continue to exist and are likely to be greater in smaller hospital systems where resources are often challenged. Respite care services such as home care, day care, or institutional care have been reported to benefit caregivers [50,52], but these are generally unavailable in rural and medically underserved communities. Social support has also been noted as an important mediator of the caregiver burden [53], whether formal support group forums provided by formal healthcare systems or informal support provided by the community, the church, or online support forums such as that polled in this study.

A limitation of this study is that it represents a sub-sample of caregivers who read and post on Reddit forums. Caregivers at risk of limited digital literacy skills may not be represented in this group, therefore other methods may be needed to better understand the caregiver burden in this population.

## 5. Conclusions

Caregivers hold an important and often hidden role in our society. Understanding their burdens and needs may provide researchers with insights into how to best support this population. The natural language processing of large social media platforms such as Reddit offers researchers a method to understand the public discourse regarding topics of interest, but it can be difficult to synthesize this information into usable themes. Combing natural language processing expertise with clinical subject matter experts allows for researchers to extract actionable information from online communities. The topics detected in this analysis are clinically helpful for supporting caregiver’s mental health and providing resources for their care. In addition, future research that ties the economic impacts of these burdens could help quantify the importance of improving the health of patient populations and thereby reducing the burdens on their caregivers. These themes help us to understand what caregivers struggle the most with and therefore can direct policymakers and clinicians to better create caregiver-specific resources to help them as they support others.

## Figures and Tables

**Table 1:** Top ten themes found in the Reddit Submission-only corpus. The percent column provides the percent of the posts in the subreddit corpus covered by the topic.

ID.	Theme	Percent
1	Wearable devices/other technological tools for patients and caregivers	0.7
2	Support groups for caregivers	4.3
3	Paid caregivers	3.9
4	Personal emotional toll on individuals especially focused on time	33.5
5	Caregiver burden specific to cancer care for parents	12.0
6	Logistical caregiver issues for parents	29.3
7	End-of-life care	6.2
8	Taking time off work to care for spouse	5.9
9	Physical equipment needs and care support	9.0
10	Activities of daily living needs and care support	2.3

**Figure A1:**
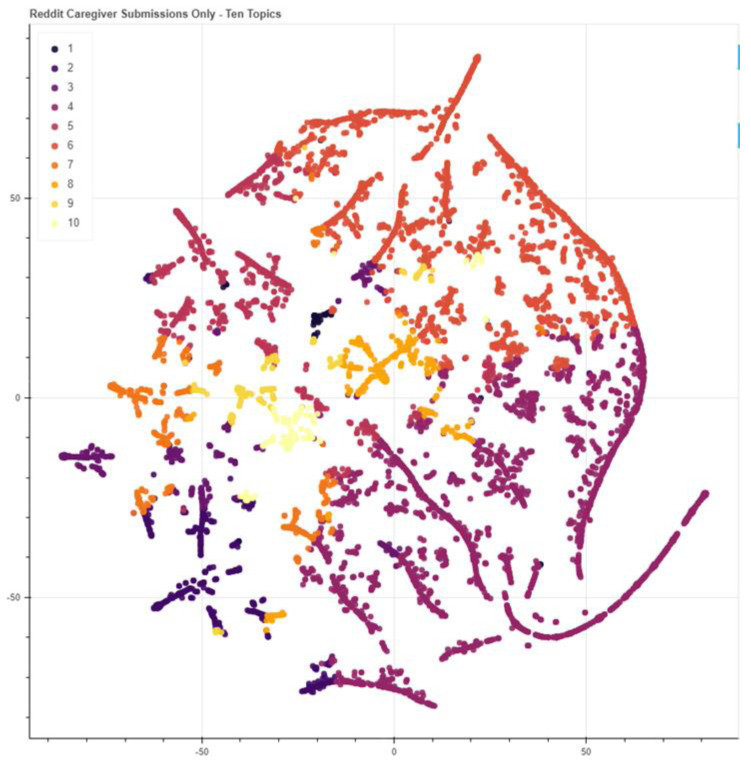
t—SNE plot of topic clusters for interactive viewing of posts. Each dot represents a Reddit submission post. Clustering or shapes represents cohesiveness in the body of the text. Colors represent topic themes (1–10). Hovering over a dot provides the associated text in the Reddit submission post allowing for quick ingestion of complex themes.

**Figure A2:**
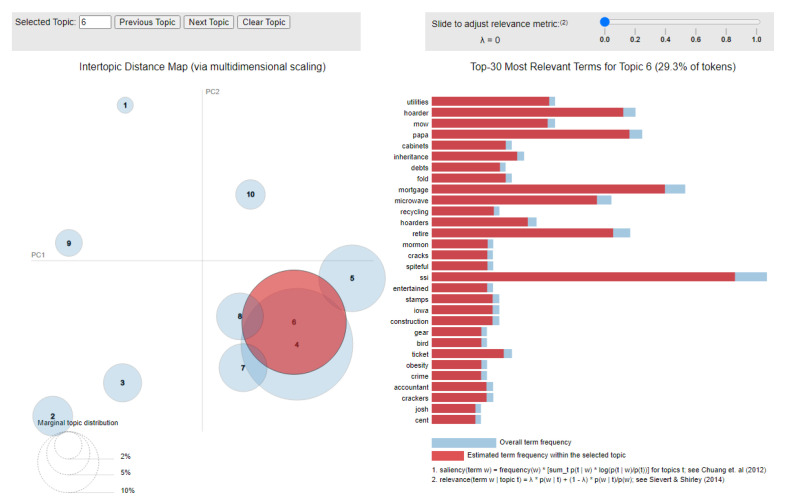
PyLDAvis interactive viz showing term frequency within corpus and topics. Topic themes are represented as circles number 1—10 and clicking on the numbered circle provides the most common terms within the topic cluster. Adjusting the lambda bar in the upper right corner providers the reviewer with the most common words in the cluster and the most common words in the cluster that are not found in the corpus [54,55].

## Data Availability

All data are publicly available via the Reddit Application Programming Interface. Data were accessed 21 June 2022 from pushshift.io using the following searchterms = [“CaregiverSupport”, “caregivers”, “caregiving”, “caregiversofreddit”, “cancercaregivers”] within this API call = “https://api.pushshift.io/reddit/search/{}/?subreddit={}&limit=10000&before=” in a loop subtracting epochs over time.
